# Impact of Sodium-Glucose Co-Transporter-2 (SGLT2) Inhibitors on Nocturnal Polyuria: A Cross-Sectional Study

**DOI:** 10.7759/cureus.80263

**Published:** 2025-03-08

**Authors:** Seisuke Sato, Takafumi Yoshimura, Kurumi Komori, Takanobu Saheki, Toshihiro Kobayashi, Kensaku Fukunaga, Hitomi Imachi, Koji Murao

**Affiliations:** 1 Internal Medicine, Sato Internal Medicine Clinic, Takamatsu, JPN; 2 Endocrinology and Metabolism, Kagawa University, Miki-chō, JPN

**Keywords:** diabetes, nocturia, nocturnal polyuria, sglt2-inhibitors, sodium-glucose cotransporter-2 (sglt-2) inhibitors, tofogliflozin

## Abstract

Aim: The aim of this study was to investigate the impact of sodium-glucose cotransporter 2 (SGLT2) inhibitors on nocturnal polyuria. Additionally, this study aimed to compare the short-acting SGLT2 inhibitor tofogliflozin and other SGLT2 inhibitors.

Methods: A questionnaire was administered to 142 patients undergoing treatment for type 2 diabetes to assess the presence of nocturnal polyuria.

Results: Among the 69 patients not taking SGLT2 inhibitors (Non-SGLT2-I Group), 41 (59.4%) reported no nocturnal polyuria, while 28 (40.6%) reported its presence. In contrast, among the 73 patients taking SGLT2 inhibitors (SGLT2-I Group), 34 (46.6%) reported no nocturnal polyuria, whereas 39 (53.4%) reported it. Further categorization of the SGLT2-I Group into those taking tofogliflozin (Tofo Group, 23 patients) and those using other SGLT2 inhibitors (Other SGLT2-I Group, 50 patients) revealed the following: In the Tofo Group, 15 (65.2%) patients reported no nocturnal polyuria, while eight (34.8%) did. In the Other SGLT2-I Group, 19 (38%) patients reported no nocturnal polyuria, while 31 (62%) reported it. A comparison between the Non-SGLT2-I and the Other SGLT2-I groups showed a significantly higher occurrence of nocturnal polyuria in the latter (p = 0.026). Furthermore, in the comparison between the Tofo Group and the Other SGLT2-I Group, the latter showed a significantly higher incidence of nocturnal polyuria (p = 0.043).

Conclusions: Tofogliflozin is less likely to exacerbate nocturnal polyuria compared to other SGLT2 inhibitors.

## Introduction

Sodium-glucose cotransporter 2 (SGLT2) inhibitors have been reported to possess not only cardioprotective but also renoprotective effects, classifying them as drugs that extend beyond the category of diabetes medications [1]. Their mechanism of action involves inhibiting SGLT2 in the proximal tubule, leading to the excretion of approximately 60 g of glucose in the urine. This process not only improves blood glucose control but also contributes to weight loss and a decrease in body fat, particularly visceral fat [2]. However, the increased urinary glucose excretion may cause frequent urination, especially nocturia. Nocturia is a condition in which an individual wakes up more than once during the night to urinate. In fact, SGLT2 inhibitors increase the number of urinations during the day and night [3]. Furthermore, the most common reason for discontinuing SGLT2 inhibitors is reported to be frequent urination [4]. It is considered important to inquire about frequent urination during consultation when using SGLT2 inhibitors for their continued use. Patients with diabetes are known to be particularly prone to nocturia [5]. In a study by Furukawa et al., it was found that 81.4% of Japanese patients with diabetes experienced nocturia, with approximately half of them voiding more than twice per night [6]. According to the Guidelines for Nocturia Treatment by the Japan Urinary Function Society, nighttime voiding exceeding two episodes interferes with restful sleep and significantly impairs quality of life (QOL) [7]. Waking up during the night to use the bathroom is associated with a reduced QOL and overall impairment in daily functioning [8]. Therefore, it is recommended that nighttime voiding be limited to fewer than two occurrences per night.

Currently, six types of SGLT2 inhibitors are available in Japan, with varied half-lives [9-14]. Among them, ipragliflozin has the longest half-life at approximately 15 hours, while the half-life of other formulations exceeds 10 hours. In contrast, tofogliflozin has a notably shorter half-life of around five hours. In previous reports, tofogliflozin was associated with a lower urinary frequency between 0:00 and 6:00 am compared to ipragliflozin. Additionally, switching from other SGLT2 inhibitors to tofogliflozin has been reported to reduce the frequency of nocturnal urination. These findings suggest that variations in half-life may impact nocturia. However, these reports are based on a limited number of cases [15,16]. Furthermore, there are no studies comparing the effects of SGLT2 inhibitors with other hypoglycemic agents on nocturia. To investigate whether these differences in half-life affect nocturia, this study was conducted to examine the nocturnal voiding frequency in patients with type 2 diabetes, comparing those using SGLT2 inhibitors with those not using them, as well as assessing differences between tofogliflozin and other SGLT2 inhibitors.

## Materials and methods

This cross-sectional study was conducted from April 1, 2020, to June 30, 2020, at Kagawa University Hospital, Marugame Medical Center, and Mitoyo Municipal Hospital, located in the Kagawa Prefecture, Japan. The study was conducted in accordance with the Declaration of Helsinki, the Ethical Guidelines for Clinical Research, and other relevant guidelines and regulations. It was approved by the Kagawa University Ethics Committee (approval number: 2023-055).

Participants

Patients with type 2 diabetes who received outpatient care were surveyed regarding their nocturnal voiding frequency. Patients undergoing treatment for urological conditions were excluded from the study. For patients using SGLT2 inhibitors, only those who had been on the medication for at least three months from the start of treatment were included. 

Data collection

Data was collected using a structured, self-administered questionnaire. The questionnaire was originally developed in Japanese. An English translation is provided in the Appendices. The questionnaire included the following question for the assessment of nocturia 'How many times do you wake up during the night to urinate?' Respondents were instructed to check one of the following options: 0 times, 1 time, 2 times, or 3 times or more. The criteria for nocturia were based on the Nocturia Treatment Guidelines, which define nocturia as the occurrence of two or more episodes of nighttime voiding, which significantly impairs QOL [7]. Individuals with one or fewer episodes were classified as not having nocturia. The primary endpoint of the study was the presence or absence of nocturia.

Data analysis

Statistical analyses were performed using EZR (Division of Hematology, Saitama Medical Center, Jichi Medical University, Saitama, Japan). EZR is a graphical user interface for R (R Foundation for Statistical Computing, Austria, Vienna). Data were presented as mean ± standard deviation (SD). Comparisons between groups were made using the Student's t-test or Fisher’s exact test. Statistical significance was defined as P < 0.05.

## Results

A total of 142 patients participated in the study. The patient demographics were as follows: mean age 70.7 ± 11.7 years, with 79 (55.6%) male patients and 63 (44.4%) female patients. BMI was 24.4 ± 3.5 kg/m^2^, glycated hemoglobin (HbA1c) level was 6.8% ± 0.7%, and estimated glomerular filtration rate (eGFR) was 70.3 ± 21.3 mL/minute/1.73 m². In addition, 74 (52.1%) patients had hypertension as a comorbidity. Among the patients, 73 were using SGLT2 inhibitors, with the following distribution: 26 (35.6%) were using ipragliflozin, 23 (31.5%) were using tofogliflozin, 15 (20.5%) were using empagliflozin, three (4.1%) were using canagliflozin, three (4.1%) were using dapagliflozin, and three (4.1%) were using luseogliflozin (Table 1).

**Table 1:** Characteristics of the study participants distributed according to the use of SGLT2 inhibitors ARB: angiotensin receptor blocker; ACE: angiotensin-converting enzyme; CCB: calcium channel blocker; GLP-IRAs: glucagon-like peptide-1 receptor agonist; DPP4: dipeptidyl peptidase 4; SGLT2: sodium-glucose co-transporter 2

Variables	Total participants (N=142)	Participants using SGLT2 inhibitors (n=73)	Participants not using SGLT2 inhibitors (n=69)	P value
Age (years), mean (SD)	70.7 (11.7)	70.4 (11.4)	71.1 (12.0)	0.726
Men, n (%)	79 (55.6)	43 (58.9)	36 (52.2)	0.5
Women, n (%)	63 (44.4)	30 (41.1)	33 (47.8)	
BMI (kg/m^2^), mean (SD)	24.4 (3.5)	24.9 (3.1)	23.8 (3.7)	0.052
HbA1c (%), mean (SD)	6.8 (0.7)	6.9 (0.6)	6.7 (0.8)	0.073
eGFR (mL/minute/1.73m^2^), mean (SD)	70.3 (21.3)	68.6 (21.9)	72.1 (20.7)	0.325
Medications, n (%)				
Antidiabetic drugs	136 (95.8)	73 (100)	63 (91.3)	0.011
DPP4 inhibitors	76 (53.5)	39 (53.4)	37 (53.6)	-
Biguanides	56 (39.4)	26 (35.6)	30 (43.4)	-
GLP-1RA	34 (23.9)	25 (34.2)	9 (13.0)	-
Sulfonylureas	22 (15.5)	15 (20.5)	7 (10.1)	-
Alpha-glucosidase inhibitors	20 (14.1)	13 (17.8)	7 (10.1)	-
Glinides	17 (12.0)	12 (16.4)	6 (8.7)	-
Pioglitazone	18 (12.7)	15 (20.5)	2 (2.9)	-
Insulin	16 (11.3)	12 (16.4)	4 (5.8)	-
Antihypertensive drug	74 (52.1)	51 (69.9)	23 (33.3)	<0.001
ARB or ACE-I	61 (43.0)	43 (58.9)	18 (26.0)	-
CCB	49 (34.5)	36 (49.3)	13 (18.8)	-
Diuretics	6 (4.2)	5 (6.8)	1 (1.4)	-
Others	9 (6.3)	7 (9.6)	2 (2.9)	-
SGLT2 inhibitors, n (%)				
Ipragliflozin	-	26 (35.6)	-	-
Tofogliflozin	-	23 (31.5)	-	-
Empagliflozin	-	15 (20.5)	-	-
Canagliflozin	-	3 (4.1)	-	-
Dapagliflozin	-	3 (4.1)	-	-
Luseogliflozin, n(%)	-	3 (4.1)	-	-
Nighttime voiding frequency, n (%)				
0 time	14 (9.8)	5 (6.8)	9 (13.0)	0.345
1 time	61 (43.0)	29 (39.7)	32 (46.4)	
2 times	55 (38.7)	33 (45.2)	22 (31.9)	
3 times or more	12 (8.5)	6 (8.2)	6 (8.7)	
Nighttime voiding 0-1 time (Not nocturia)	75 (52.8)	34 (46.6)	41 (59.4)	0.134
Nighttime voiding ≥ 2 times (Nocturia)	67 (47.2)	39 (53.4)	28 (40.6)	

Among the 142 patients, the distribution of nocturnal voiding frequency was as follows: 14 (9.8%) reported zero episodes, 61 (43%) experienced one episode, 55 (38.7%) reported two episodes, and 12 (8.5%) reported three or more episodes. Nocturia was absent in 75 (52.8%) patients, while 67 (47.2%) patients experienced it. In the non-SGLT2 inhibitor group (non-SGLT2-I Group) (n=69), nine (13%) reported zero episodes, 32 (46.4%) reported one episode, 22 (31.9%) reported two episodes, and six (8.7%) reported three or more episodes. Nocturia was absent in 41 (59.4%) patients, while 28 (40.6%) patients experienced it. In the SGLT2-I Group (n=73), five (6.8%) patients reported zero episodes, 29 (39.7%) experienced one episode, 33 (45.2%) experienced two episodes, and six (8.2%) experienced three or more episodes. Nocturia was absent in 34 (46.6%) patients, while 39 (53.4%) patients reported experiencing it.

**Figure 1:**
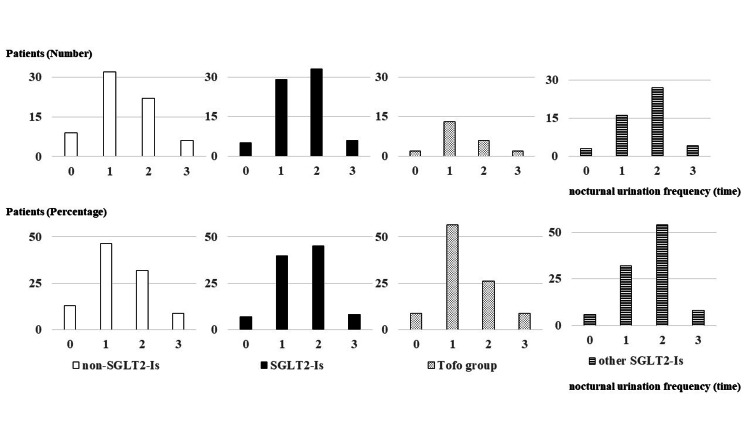
Nocturnal voiding frequency among participants (N=142) The upper panel presents the actual nocturnal urination frequency in participants not using SGLT2 inhibitors (non-SGLT-I Group; n=69), using SGLT2 inhibitors (SGLT2-I Group; n=73), using tofogliflozin (Tofo Group; n=23), and using other SGLT2 inhibitors (Other SGLT2-I Group; n=50), while the lower panel shows the percentage of patients in each group. The Tofo group exhibits a similar trend as the non-SGLT-I Group. SGLT2: sodium-glucose co-transporter 2

**Figure 2:**
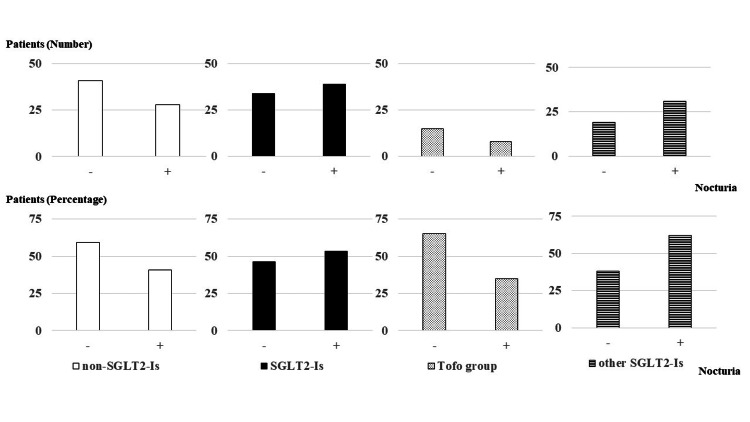
Actual occurences of nocturia in the participants (N=142) The upper panel depicts the actual occurrences of nocturnal urination in participants not using SGLT2 inhibitors (non-SGLT-I Group; n=69), using SGLT2 inhibitors (SGLT2-I Group; n=73), using tofogliflozin (Tofo Group; n=23), and using other SGLT2 inhibitors (Other SGLT2-I Group; n=50), while the lower panel shows the percentage of patients in each group. The Tofo group exhibits a similar trend as the non-SGLT-I Group. SGLT2: sodium-glucose co-transporter 2

When SGLT2 inhibitors were categorized into the tofogliflozin group (Tofo Group) and the other SGLT2 inhibitors group (Other SGLT2-I Group), the Tofo group consisted of 23 participants. The distribution of nocturnal voiding frequency in the Tofo group was as follows: two (8.7%) patients reported zero episodes, 13 (56.5%) reported one episode, six (26%) reported two episodes, and two (8.7%) reported three or more episodes. Overall, nocturia was absent in 15 (65.2%) patients and present in eight (34.8%) patients. In the Other SGLT2-I Group (n=50), the distribution of nocturnal voiding frequency was as follows: three (6%) patients reported zero episodes, 16 (32%) reported one episode, 27 (54%) reported two episodes, and four (8%) reported three or more episodes. Overall, nocturia was absent in 19 (38%) patients and present in 31 (62%) patients (Table 2, Figures 1, 2).

**Table 2:** Comparision of the characteristics of participants using tofogliflozin and those using other SGLT2 inihibitors ARB: angiotensin receptor blocker; ACE: angiotensin-converting enzyme; CCB: calcium channel blocker; GLP-IRAs: glucagon-like peptide-1 receptor agonist; DPP4: dipeptidyl peptidase 4; SGLT2: sodium-glucose co-transporter 2

Variables	Participants using tofogliflozin (n=23)	Participants using other SGLT2 inhibitors (n=50)	P value
Age (years), mean (SD)	74.5 (9.6)	68.5 (11.8)	0.038
Men, n (%)	13 (56.5)	30 (60)	0.803
women, n (%)	10 (43.4)	20 (40)	
BMI, mean (SD)	25.0 (3.6)	24.9 (2.9)	0.882
HbA1c (%), mean (SD)	6.8 (0.4)	7.0 (0.7)	0.152
eGFR (mL/min/1.73m2)	65.3 (23.0)	70.1 (21.4)	0.384
Medications, n (%)			
Concomitant antidiabetic drug	23 (100)	50 (100)	-
DPP4 inhibitors	10 (43.4)	29 (58)	-
Biguanides	6 (26.1)	20 (40)	-
GLP-1RAs	10 (43.4)	15 (30)	-
Sulfonylureas	3 (13.0)	12 (24)	-
a-glucosidase inhibitors	5 (21.7)	8 (16)	-
Glinides	6 (26.1)	9 (18)	-
Pioglitazone	4 (17.4)	11 (22)	-
Insulin	4 (17.4)	8 (16)	-
antihypertensive drug	16 (69.6)	35 (70)	1
ARB or ACE-I	15 (65.2)	28 (56)	-
CCB	10 (43.4)	26 (52)	-
Diuretics	1 (4.3)	4 (8)	-
others	1 (4.3)	6 (12)	-
Nighttime voiding frequency, n (%)			
0 times	2 (8.7)	3 (6)	0.112
1 time	13 (56.5)	16 (32)	
2 times	6 (26)	27 (54)	
3 times or more	2 (8.7)	4 (8)	
Nighttime voiding 0-1 times (not nocturia)	15 (65.2)	19 (38)	0.043
Nighttime voiding ≥ 2 times (Nocturia)	8 (34.8)	31 (62)	

There was no significant difference in nocturia based on the presence or absence of SGLT2 inhibitor use. However, when comparing the Non-SGLT2-I Group and the Other SGLT2-I Group, a significant increase in nocturia was observed in the Other SGLT2-I group (p = 0.026; Table 3).

**Table 3:** Comparison between the characteristics of participants using SGLT2 inhibitors other than tofogliflozin (Other SGLT2-I Group) and those not using SGLT2 inhibitors (Non-SGLT2-I Group) SGLT2: sodium-glucose co-transporter 2

Variables	Other SGLT2-I Group (n=50)	Non-SGLT2-I Group (n=69)	P value
Age (years), mean (SD)	68.5 (11.8)	71.1 (12.0)	0.25
Men, n (%)	30 (60)	36 (52.2)	0.457
Women, n (%)	20 (40)	33 (47.8)	
BMI, mean (SD)	24.9 (2.9)	23.8 (3.7)	0.088
HbA1c (%), mean (SD)	7.0 (0.7)	6.7 (0.8)	0.045
eGFR (mL/minute/1.73m^2^), mean (SD)	70.1 (21.4)	72.1 (20.7)	0.607
Medications, n (%)			
Antidiabetic drug	50 (100)	63 (91.3)	0.039
DPP4-Is	29 (58)	37 (53.6)	-
Biguanides	20 (40)	30 (43.4)	-
GLP-1RAs	15 (30)	9 (13.0)	-
Sulfonylureas	12 (24)	7 (10.1)	-
Alpha-glucosidase inhibitors	8 (16)	7 (10.1)	-
Glinides	9 (18)	6 (8.7)	-
Pioglitazone	11 (22)	2 (2.9)	-
Insulin	8 (16)	4 (5.8)	-
antihypertensive drug	35 (70)	23 (33.3)	<0.001
ARB or ACE-I	28 (56)	18 (26.0)	-
CCB	26 (52)	13 (18.8)	-
Diuretics	4 (8)	1 (1.4)	-
others	6 (12)	2 (2.9)	-
Nighttime voiding frequency, n (%)			
0 times	3 (6)	9 (13.0)	0.101
1 time	16 (32)	32 (46.4)	
2 times	27 (54)	22 (31.9)	
3 times or more	4 (8)	6 (8.7)	
Nighttime voiding 0-1 time (Not Nocturia)	19 (38)	41 (59.4)	0.026
Nighttime voiding ≥ 2 times (Nocturia)	31 (62)	28 (40.6)	

Furthermore, when comparing the Tofo Group and the Other SGLT2-I Group, a significant increase in nocturia was also observed in the latter (p = 0.043). No significant differences were observed between the Tofo Group and the Other SGLT2-I Group in terms of parameters such as weight, HbA1c, and renal function.

Throughout the study, no apparent adverse events, including urinary tract infections, were observed in any of the groups.

## Discussion

In this study, approximately half of the patients with diabetes experienced nocturia. Diabetes can contribute to various lower urinary tract dysfunctions such as a decreased urinary stream, urinary retention, delayed urination, and abdominal pressure urination [17]. Additionally, storage urinary dysfunctions, including frequent urination, urgency, and urinary incontinence, are also observed. According to a report from Japan, among patients with type 2 diabetes with an average age of 60.8 years, urinary dysfunctions occurred at a frequency ranging from 38% to 71%, while storage urinary dysfunctions were observed at a frequency ranging from 38% to 55% [18]. These findings are consistent with the results of our current study.

The mechanism underlying nocturia appears to be osmotic diuresis due to high blood glucose levels. Given their mechanism of action, SGLT2 inhibitors were expected to significantly increase nighttime urinary frequency. However, in this study, no statistically significant difference was observed in nighttime urinary frequency between patients using and not using SGLT2 inhibitors. Nonetheless, when comparing other SGLT2 inhibitors to non-SGLT2 inhibitors, a notable increase in nocturia was observed in the participants using SGLT2 inhibitors other than tofogliflozin. Furthermore, when comparing the group using tofogliflozin and the group using other SGLT2 inhibitors, nocturia was significantly more prevalent in the latter. Among those not taking SGLT2 inhibitors, patients with a single nocturnal void were the most prevalent, while SGLT2 inhibitor users predominantly experienced two nocturnal voids. However, upon further analysis of SGLT2 inhibitors, it was observed that in patients using SGLT2 inhibitors other than tofogliflozin, the most common occurrence was two nocturnal voids, whereas, in the participants using tofogliflozin, a single nocturnal void was the most prevalent. This finding suggests that SGLT2 inhibitors, except for tofogliflozin, have a half-life exceeding 10 hours, resulting in continuous nighttime urinary glucose excretion when taken in the morning, thus increasing nighttime urine volume. However, tofogliflozin, with a significantly shorter half-life of 5.4 hours, likely loses its effect during sleep, resulting in minimal impact on nighttime urine volume.

While this characteristic of tofogliflozin is advantageous in terms of reducing nighttime urination frequency, concerns have been raised about its potentially weaker blood glucose-lowering effect compared to other medications owing to the absence of nighttime urinary glucose excretion. A review of the package inserts for each product indicates that urinary glucose excretion after a single dose in patients with type 2 diabetes who had normal renal function was approximately 71 g for ipragliflozin 100 mg, 74 g for tofogliflozin 20 mg, 75 g for empagliflozin 10 mg, 86 g for canagliflozin 100 mg, and 88 g for luseogliflozin 2.5 mg [19-22]. No baseline change was provided for dapagliflozin, rendering its urinary glucose excretion uncertain [23]. Overall, urinary glucose excretion for these medications ranged from 70 g to 90 g. Notably, a previous report did not indicate a significant difference in urinary glucose excretion between ipragliflozin and tofogliflozin, suggesting that the duration of action and urinary glucose excretion may not necessarily be correlated.

A clinical trial by Kawaguchi et al. comparing ipragliflozin and tofogliflozin used postprandial blood glucose levels, confirmed by flash glucose monitoring, as the primary outcome measure, along with time below range (TBR) [24]. In their study, the tofogliflozin group demonstrated significantly lower blood glucose levels and TBR. Urinary glucose excretion was also examined as a secondary outcome. Although there was no significant difference in the total 24-hour urinary glucose excretion between the two groups, the tofogliflozin group exhibited higher urinary glucose excretion from 8 a.m. to 10 p.m., while the ipragliflozin group showed higher urinary glucose excretion from 10 p.m. to 8 a.m. These results suggest that despite its shorter duration of action, tofogliflozin’s urinary glucose excretion and blood glucose-lowering effects are comparable to those of other SGLT2 inhibitors. Additionally, its lower nighttime urinary glucose excretion may help reduce the risk of nocturnal hypoglycemia.

Various hormones are secreted during hypoglycemia to raise blood glucose levels, with adrenaline being the primary counterregulatory hormone. However, during nighttime sleep, the autonomic nervous system becomes predominantly parasympathetic, leading to decreased compensatory adrenaline secretion in response to low blood glucose levels, increasing the risk of unconscious hypoglycemia [25]. Additionally, elderly individuals may be particularly susceptible to age-related autonomic dysfunction, which can impair the ability to recognize warning symptoms of hypoglycemia [26]. Patients with type 2 diabetes experience an increase in hypoglycemic events as they age, regardless of the duration of their condition [27]. Moreover, hypoglycemia is linked to a heightened risk of falls and fractures [28]. Therefore, when prescribing SGLT2 inhibitors that remain active during the night, it is important to monitor not only for frequent urination but also for hypoglycemia. Moreover, nocturnal polyuria not only reduces the quality of life by causing reduced daytime activity due to sleep deprivation but also increases the risk of fractures, particularly in older adults. An observational study involving 784 Japanese individuals aged 70 years or older reported that nocturnal urination with a frequency of two or more significantly increased the risk of falls and reduced overall survival [29]. For these reasons, nocturnal polyuria should be considered a critical symptom in the management of elderly patients with diabetes. Tofogliflozin, which has reduced nighttime efficacy, not only does not worsen nocturnal polyuria but also has the potential to prevent nighttime hypoglycemia and reduce the risk of fractures, making it a preferable SGLT2 inhibitor for elderly patients.

When evaluating nocturnal polyuria, it is important to consider that conditions other than diabetes, such as overactive bladder and prostate enlargement, may also be involved [30]. Therefore, it is advisable to thoroughly investigate the presence of other diseases before attributing nocturnal polyuria solely to diabetes or its treatments. Additionally, a key limitation of this study is that many patients who complained of nocturnal polyuria were already using tofogliflozin before the investigation, which could potentially introduce bias. Factors contributing to other causes of nocturnal frequency have not been examined, and the evaluation of nocturnal frequency was based solely on a questionnaire, as bladder diaries and nocturnal urine volume measurements were not conducted, which constitutes a limitation.

## Conclusions

In patients with type 2 diabetes taking SGLT2 inhibitors orally, there is a tendency for nocturnal polyuria, especially in those using SGLT2 inhibitors other than tofogliflozin, where nocturnal polyuria was significantly more frequent compared to patients not taking SGLT2 inhibitors. Conversely, in participants using tofogliflozin, nocturnal polyuria was significantly lower compared to the participants taking other SGLT2 inhibitors. These findings suggest that tofogliflozin may be effective not only in reducing nocturnal urination but also in reducing the risk of nighttime hypoglycemia and fractures.

## Appendices

Questionnaire on nocturnal enuresis

Age:

Sex:

Do you go to a urologist for medication?

□ Yes　□ No

Are you on medication for high blood pressure?

□ Yes　□ No

If you are treating hypertension, what is the name of your medication?

(　　　　　　　　　　　　　　　　　　　　　　　　　　　　)

How many times do you wake up during the night to urinate?"

□ 0 times　□ 1 time　□ 2 times　□ 3 times or more.
